# *ZEB2,* a new candidate gene for asplenia

**DOI:** 10.1186/1750-1172-9-2

**Published:** 2014-01-08

**Authors:** Linda Pons, Sophie Dupuis-Girod, Marie-Pierre Cordier, Patrick Edery, Massimiliano Rossi

**Affiliations:** 1 Hospices Civils de Lyon, Groupe Hospitalier Est, Service de Génétique et Centre de référence des anomalies du développement, Bron F-69677, France; 2 Centre de recherche en Neurosciences de Lyon, Inserm U1028, CNRS UMR 5292, UCBL, équipe TIGER, Bron, France

**Keywords:** Mowat-Wilson syndrome, *ZEB2*, Asplenia

## Abstract

Primary asplenia is a rare condition with poorly known etiology. Mowat-Wilson syndrome (MWS) is characterized by typical facial dysmorphisms, intellectual disability, microcephaly, epilepsy and the possible presence of internal organ malformations. It is caused by heterozygous mutations or deletions in the *ZEB2* gene. Nearly 180 patients have been reported to date, but only one with asplenia. We report here spleen hypo/aplasia in 4 out of 6 MWS patients, with severe infectious complications for 3 of them. Our report shows that spleen hypo/aplasia is part of the MWS phenotype and makes *ZEB2* a possible candidate gene for primary asplenia.

## Letters to the editor

Primary congenital asplenia is a rare condition with poorly known genetic bases. It can be part of multiple congenital abnormalities syndromes or it can be isolated, which is extremely rare with about 70 patients reported to date [1]. Recently Bolze et al. identified heterozygous mutations in the *RPSA* gene in more than half the patients studied with isolated congenital asplenia [2]. Mowat-Wilson syndrome (MWS, OMIM #235730) is characterized by typical facial features (large medially sparse eyebrows, hypertelorism, deep set eyes, uplifted ear lobes with central depression, saddle nose and a pointed chin), intellectual disability, microcephaly, epilepsy and congenital malformations including Hirschsprung disease, genito-urinary abnormalities, cardiac defects, corpus callosum agenesis and ocular anomalies [3]. This syndrome was first described in 1998 [4] and is caused by heterozygous mutations or deletions in the Zinc finger E-box-binding homeobox 2 gene (*ZEB2)*[5,6]. Nearly 180 patients have been reported to date. Asplenia was reported in only one case [7]. Interestingly, it has been shown that *ZEB2* has a diffuse expression in several mouse and human organs, including the spleen [8,9].

We report here spleen hypo/aplasia in 4 out of 6 unrelated MWS patients referred to our genetic department, with severe infectious complications for 3 of them.

The first patient is a female born to unrelated parents, presenting with a typical facies, microcephaly, postnatal short stature, developmental delay, corpus callosum agenesis, ventricular septal defect, strabismus and left dimmed vision. A *de novo* c.2083C > T heterozygous mutation of the *ZEB2* gene was identified, thus confirming the diagnosis. At the age of 8 months, she suffered from purpura fulminans related to a severe *Streptococcus Pneumoniae* infection (serotype 12 F) with severe necrosis sequellae including the loss of 5 toes and the right heel requiring a skin graft. Asplenia was diagnosed on ultrasound scan (USS).

The second patient is a female born to unrelated parents. Typical dysmorphic features, microcephaly, developmental delay, epilepsy, corpus callosum agenesis, ventricular septal defect and patent ductus arteriosus, club foot and strabismus were consistent with the diagnosis of MWS. A *de novo* c.600_640dup heterozygous mutation of the *ZEB2* gene was identified. At the age of 1 year, she developed meningitis related to *Streptococcus Pneumoniae* infection (serotype 17 F on cerebrospinal fluid culture) complicated by moderate intracranial hypertension. USS revealed asplenia.

The third patient is a female born to unrelated parents. She had typical facial dysmorphisms, severe intellectual disability, microcephaly, epilepsy, postnatal short stature, ventricular septal defect, vesico-ureteric reflux, club foot and unilateral choanal atresia. Molecular analysis of the *ZEB2* gene showed a *de novo* c.1762G > T heterozygous mutation*.* She had two pneumococcal septicemias at the ages of 2 and 3 years. Abdominal USS revealed severe splenic hypoplasia (main axis: 34 mm, average for weight: 80 mm, range 78–87 mm). Howell-Jolly bodies were absent.

The fourth patient is a female born to non-related parents, presenting with a typical facies, microcephaly, postnatal short stature, developmental delay, epilepsy, atrial septal defect and patent ductus arteriosus, strabismus. A *de novo* c.2761C > T heterozygous mutation of the *ZEB2* gene was identified confirming the diagnosis of MWS. She had no severe infections. USS showed moderate splenic hypoplasia (main axis: 46 mm, average for weight: 78 mm, range 76–78 mm).

The 2 other MWS patients followed in our genetic department had a normal spleen on USS and did not have severe infections.

On one hand, our report shows that spleen hypo/aplasia is a part of the phenotype of MWS. USS should be systematically performed on MWS patients in order to rule out spleen hypo/aplasia because of potential complications’ severity. Prevention of severe infections in cases of asplenia or severe hypoplasia, effectively requires appropriate antibiotic prophylaxis and vaccination that can restore the pool of memory B cells [10].

As our cohort only includes 6 MWS subjects, we think that other studies should be done to confirm if asplenia/ spleen hypoplasia is a new feature of this multisystem disorder.

Indeed, the case of asplenia previously reported has a *de novo* c.696C > G heterozygous mutation of *ZEB2*, so a different one from our patients. Furthermore, no spleen hypo/aplasia has been described in previously reported patients with *de novo* c.2083C > T or c.2761C > T heterozygous mutations of *ZEB2* found in patients 1 and 4 [11].

On the other hand, this report emphasizes the potential role of *ZEB2* in human spleen development. This association makes this gene a possible new candidate for isolated congenital asplenia. Indeed all cases cannot be attributed to *RPSA* gene, suggesting a possible genetic heterogeneity. Although mutations of *ZEB2* are generally associated with much more complex conditions, it cannot be excluded that some missense mutations could be responsible for apparently isolated asplenia [2].
